# Drug survival of IL-12/23, IL-17 and IL-23 inhibitors for moderate-to-severe plaque psoriasis: a retrospective multicenter real-world experience on 5932 treatment courses – IL PSO (Italian landscape psoriasis)

**DOI:** 10.3389/fimmu.2023.1341708

**Published:** 2024-01-11

**Authors:** Luigi Gargiulo, Luciano Ibba, Piergiorgio Malagoli, Anna Balato, Federico Bardazzi, Martina Burlando, Carlo G. Carrera, Giovanni Damiani, Paolo Dapavo, Valentina Dini, Francesca M. Gaiani, Giampiero Girolomoni, Claudio Guarneri, Claudia Lasagni, Francesco Loconsole, Angelo V. Marzano, Matteo Megna, Santo R. Mercuri, Massimo Travaglini, Antonio Costanzo, Alessandra Narcisi

**Affiliations:** ^1^ Dermatology Unit, IRCCS Humanitas Research Hospital, Milan, Italy; ^2^ Department of Biomedical Sciences, Humanitas University, Milan, Italy; ^3^ Department of Dermatology, Dermatology Unit, Azienda Ospedaliera San Donato Milanese, Milan, Italy; ^4^ Dermatology Unit, University of Campania L. Vanvitelli, Naples, Italy; ^5^ Dermatology Unit, IRCCS Azienda Ospedaliero-Universitaria di Bologna, Policlinico S. Orsola-Malpighi, Bologna, Italy; ^6^ Section of Dermatology, Department of Health Sciences (DISSAL), IRCCS San Martino University Hospital, Genoa, Italy; ^7^ Dermatology Unit, Fondazione IRCCS Ca’ Granda Ospedale Maggiore Policlinico, Milan, Italy; ^8^ Department of Biomedical, Surgical and Dental Sciences, University of Milan, Milan, Italy; ^9^ Dermatology and Cosmetology Unit, IRCCS San Raffaele Hospital, Milan, Italy; ^10^ Department of Biomedical Science and Human Oncology, Second Dermatologic Clinic, University of Turin, Turin, Italy; ^11^ Dermatology Unit, Department of Clinical and Experimental Medicine, Ospedale Santa Chiara, Pisa, Italy; ^12^ Department of Medicine, Section of Dermatology and Venereology, University of Verona, Verona, Italy; ^13^ Department of Biomedical and Dental Sciences and Morphofunctional Imaging, University of Messina, Messina, Italy; ^14^ Dermatological Clinic, Department of Specialized Medicine, University of Modena, Modena, Italy; ^15^ Department of Dermatology, University of Bari, Bari, Italy; ^16^ Department of Pathophysiology and Transplantation, Università degli Studi di Milano, Milan, Italy; ^17^ Section of Dermatology, Department of Clinical Medicine and Surgery, University of Naples Federico II, Naples, Italy; ^18^ Dermatology Unit, Università Vita-Salute San Raffaele, Milan, Italy; ^19^ U.O.S.D. Dermatologica - Centro per la Cura della Psoriasi, Ospedale Perrino, Brindisi, Italy

**Keywords:** IL-inhibitors, immunomodulatory therapies, inflammatory skin diseases, psoriasis, psoriasis treatment

## Abstract

**Introduction:**

The development of several effective biological drugs for moderate-to-severe plaque psoriasis has dramatically changed the lives of patients. Despite the wide use of interleukin (IL) inhibitors, limited data are available to date regarding long-term treatment persistence.

**Method:**

This multicenter retrospective real-world study evaluated 5932 treatment courses across 5300 patients, all treated with interleukin inhibitors. Drug survival was expressed by using the Kaplan-Meier estimator for each biological drug at 6, 12, 24, 36 and 48 months. We also stratified by discontinuation associated with primary or secondary ineffectiveness.

**Results:**

In our study, the most prescribed drugs were secukinumab (1412), ixekizumab (1183), and risankizumab (977). After four years of follow-up, risankizumab emerged as the treatment with the highest drug survival overall, as 91.6% of patients were still on treatment. The overall probability of drug survival at four years was comparable for tildrakizumab (83.5%), ixekizumab (82.6%), guselkumab (82.4%) and brodalumab (81.8%). When evaluating only patients who discontinued the treatment because of ineffectiveness, once again risankizumab was the molecule with the highest drug survival at 4 years (93.4%), this time followed by ixekizumab (87%). Our study, in which all IL inhibitors were adequately represented, confirmed a slightly better treatment persistence for IL-23 inhibitors, consistent with other real-world studies.

**Conclusion:**

Our experience showed that IL-23 inhibitors, and risankizumab in particular, had a higher probability of drug survival overall during a 4-year follow-up. Risankizumab and ixekizumab were less likely to be discontinued because of ineffectiveness after four years.

## 1 Introduction

Psoriasis is a chronic immune-mediated disease affecting up to 3% of the population worldwide (1). It is a condition that primarily affects the skin and joints and can severely impact patients’ quality of life and their productivity. The development of different biological treatments has dramatically changed the lives of patients affected by moderate-to-severe plaque psoriasis (1). In Italy, several biological drugs are approved for the treatment of these patients. In particular, interleukin (IL) inhibitors include drugs targeting IL-12/23 (ustekinumab), IL-17 (secukinumab, ixekizumab, brodalumab and bimekizumab) and IL-23 (guselkumab, risankizumab and tildrakizumab). These molecules have shown high efficacy and safety profiles in both clinical trials and real-world experiences, supporting the wide use of biologics in wide cohorts of patients with moderate-to-severe plaque psoriasis (2). Currently, the switch among biologics is becoming more common due to several reasons, including ineffectiveness and adverse events. Patients’ preferences also play a role in the decision to switch treatments. Because of that, it is crucial to evaluate the drug survival of these molecules since it seems to correlate with clinical response and tolerability in a real-world setting. This is particularly important if we consider that it has been shown that bio-naïve patients and those with a very short disease duration (less than two years) are more likely to have better clinical response to some molecules (3, 4). Hence, it is crucial to choose the best possible drug for each patient in order to achieve better outcomes. Current Guidelines do not give any specific recommendations regarding which biological drug should be used as a first-line treatment for each type of patient in terms of disease severity, involvement of difficult-to-treat areas, or presence of cardiometabolic comorbidities (5). Italian Guidelines provide limited evidence for the use of specific classes of biologics for some patients’ subpopulations. As a matter of fact, patients with concomitant psoriatic arthritis (PsA), should be treated with anti-TNF-alfa or anti-IL-17 drugs, while those with a diagnosis or medical history of inflammatory bowel disease should not receive IL-17 inhibitors. Regarding all other medical conditions, no strong recommendations are currently available (6). For all these reasons, during the last years, a few studies on treatment persistence have been published, showing a higher drug survival for patients treated with IL-23 inhibitors (7–10). However, limited data are available in particular for the most recently approved treatments, such as tildrakizumab and risankizumab. More research is needed to evaluate these drugs’ effectiveness and safety profiles in a real-world setting.

## 2 Method

We conducted a retrospective real-world study to assess the drug survival of IL-inhibitors in plaque psoriasis, across 5300 patients and 5932 treatment courses, with a 4-year follow-up. Patients were followed from 1^st^ January 2012 to 31^st^ December 2022 at 15 Italian Dermatology Units. Patients who discontinued a drug and started another treatment were included in the analysis as a new treatment course with a re-evaluation of all baseline characteristics. Drug survival was expressed by using the Kaplan-Meier estimator for each biological drug at 6, 12, 24, 36 and 48 months. We also stratified by discontinuation associated with primary or secondary ineffectiveness. The date of the event was defined as the date the patient discontinued the biologic for any reason. Time data was censored for patients who were still on treatment when the study was conducted. We also used the log-ranked test to assess the differences in drug survival between the subgroups. To describe demographic characteristics, we used mean and Standard Deviation (SD) for continuous variables and absolute frequency and percentage for categorical parameters. STATA/SE 17.0 software was used to conduct the data analysis and to generate graphs. For this retrospective study, institutional review board approval was waived as the study protocol did not deviate from routine clinical practice. In this study, we did not perform any procedure differently from routine clinical practice. All patients included in the study had provided written consent for retrospective analysis of anonymous data collected during routine clinical practice, including demographics and clinical severity scores. This study was conducted in accordance with the Helsinki Declaration of 1964 and its later amendments.

## 3 Results

The demographic characteristics of our cohort at the start of the treatment are available in **Table 1**.

**Table 1:** Characteristics of our population at the start of the start of the treatment.

Total patients	5300
Treatment courses	5932
	**Mean ± SD**
Age, years	53.74 ± 14.85
BMI, kg/m2	27.31 ± 5.29
PASI at Baseline	14.42 ± 7.08
	**N (%)**
Male	3898 (65.71)
PsA	1330 (22.42)
CMD	2919 (49.21)
Difficult-to-treat Areas	2716 (45.79)
Bio-Naïve	2744 (46.26)
*Current Biological Drug*	
Ustekinumab	347 (5.85)
Brodalumab	676 (11.40)
Secukinumab	1412 (23.80)
Ixekizumab	1183 (19.94)
Tildrakizumab	488 (8.23)
Risankizumab	977 (16.47)
Guselkumab	849 (14.31)

BMI, Body Mass Index; PsA, Psoriatic arthritis; PASI, Psoriasis Area and Severity Index; CMD, cardiometabolic diseases; SD, Standard Deviation.

Three thousand eight-hundred and ninety-eight patients were males (65.71%), with a mean age of 53.74 years (SD 14.85). The vast majority of our patients were Caucasians, and no other ethnicity was significantly represented in our cohort. A concomitant psoriatic arthritis (PsA) was diagnosed by rheumatologists in 1330 patients (22.42%), according to CASPAR (ClASsification of Psoriatic ARthritis) classification criteria (11). Two thousand nine hundred and nineteen patients (49.21%) presented with at least one cardio-metabolic comorbidity (including obesity, arterial hypertension, hypercholesterolemia, type II diabetes mellitus and cardiovascular diseases). At baseline, our patients had a mean body mass index (BMI) of 27.31 (5.29). The mean Psoriasis Area and Severity Index (PASI) at the start of the biological treatment was 14.42 (7.08), comparable with other real-world experiences. Two thousand seven hundred and sixteen patients (45.79%) had the involvement of at least one difficult-to-treat area (including scalp, palms/soles, nails and genitalia). Slightly less than half of our patients were naïve to biological treatments (46.26%). Regarding current treatment courses, the most common drug was secukinumab (1412 [23.80%]), followed by ixekizumab (1183 [19.44%]), risankizumab (977 [16.47%]), guselkumab (849 [14.31%]), brodalumab (676 [11.40%]), tildrakizumab (488 [8.23%]) and ustekinumab (347 [5.85%]). One thousand and fifty-one patients discontinued the biological therapy during the observation period. The causes of treatment discontinuation were: primary ineffectiveness (182 treatment courses, 3.07%), loss of effectiveness (660, 11.29%), treatment-emerging adverse events (60, 1.01%), patient’s decision (17, 0.29%) and loss of follow up (138, 2.33%). The baseline characteristics of our patients, stratified by biological treatment, are shown in **Table 2**. Remarkable differences among the biological cohorts included the proportion of bio-naïve patients (ustekinumab 17.87%; guselkumab 30.15%; brodalumab 47.78%; ixekizumab 49.28%; secukinumab 49.93%; risankizumab 50.46%; tildrakizumab 65.98%) and the proportion of patients with PsA (tildrakizumab 12.70%; risankizumab 13.61%; ustekinumab 17%; brodalumab 17.46%; guselkumab 19.20%; secukinumab 30.03%; ixekizumab 31.36%).

**Table 2:** Baseline characteristics of our population separated by biological treatment.

	Brodalumab	Ixekizumab	Secukinumab	Guselkumab	Risankizumab	Tildrakizumab	Ustekinumab
Total	676	1183	1412	849	977	488	347
Mean ± SD							
Age, years	54.22 ± 15.24	53.04 ± 14.65	54.71 ± 14.30	51.84 ± 14.98	52.40 ± 14.79	56.03 ± 15.75	56.51 ± 14.40
BMI, kg/m2	26.85 ± 5.03	27.48 ± 5.25	27.06 ± 4.81	27.26 ± 5.46	27.73 ± 5.69	26.82 ± 5.00	28.13 ± 6.16
PASI at Baseline	14.95 ± 7.30	15.49 ± 7.19	14.80 ± 6.23	12.50 ± 7.54	15.47 ± 7.30	12.64 ± 6.06	12.11 ± 7.26
N (%)							
Male	469 (69.38)	762 (64.41)	914 (64.73)	552 (65.02)	669 (68.47)	311 (63.73)	221 (63.69)
PsA	118 (17.46)	371 (31.36)	424 (30.03)	163 (19.20)	133 (13.61)	62 (12.70)	59 (17.00)
CMD	317 (46.89)	548 (46.32)	666 (47.17)	419 (49.35)	525 (53.74)	246 (50.41)	198 (57.06)
Difficult-to-treat Areas	245 (36.24)	578 (48.86)	617 (43.70)	480 (56.54)	357 (36.54)	206 (42.21)	233 (67.15)
Bio-Naïve	323 (47.78)	583 (49.28)	705 (49.93)	256 (30.15)	493 (50.46)	322 (65.98)	62 (17.87)

BMI, Body Mass Index; PsA, Psoriatic arthritis; PASI, Psoriasis Area and Severity Index; CMD, cardiometabolic diseases; SD, Standard Deviation.

**Figure 1:**
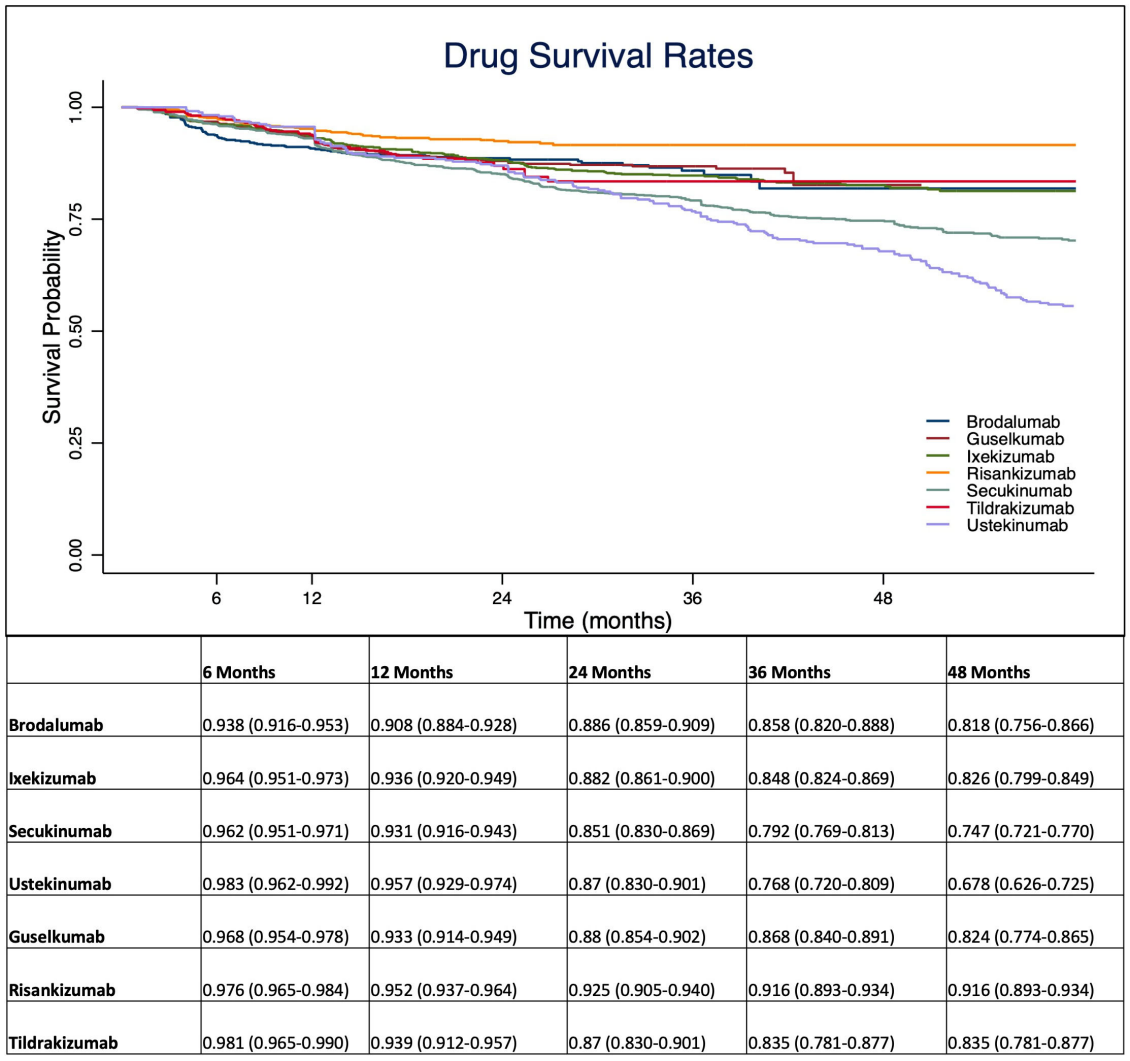
Overall probability of drug survival throughout the study period for interleukin (IL-)12/23, IL-17 and IL-23 inhibitors. Data are presented as probability with 95% C.I. (Confidence Interval).

**Figure 2:**
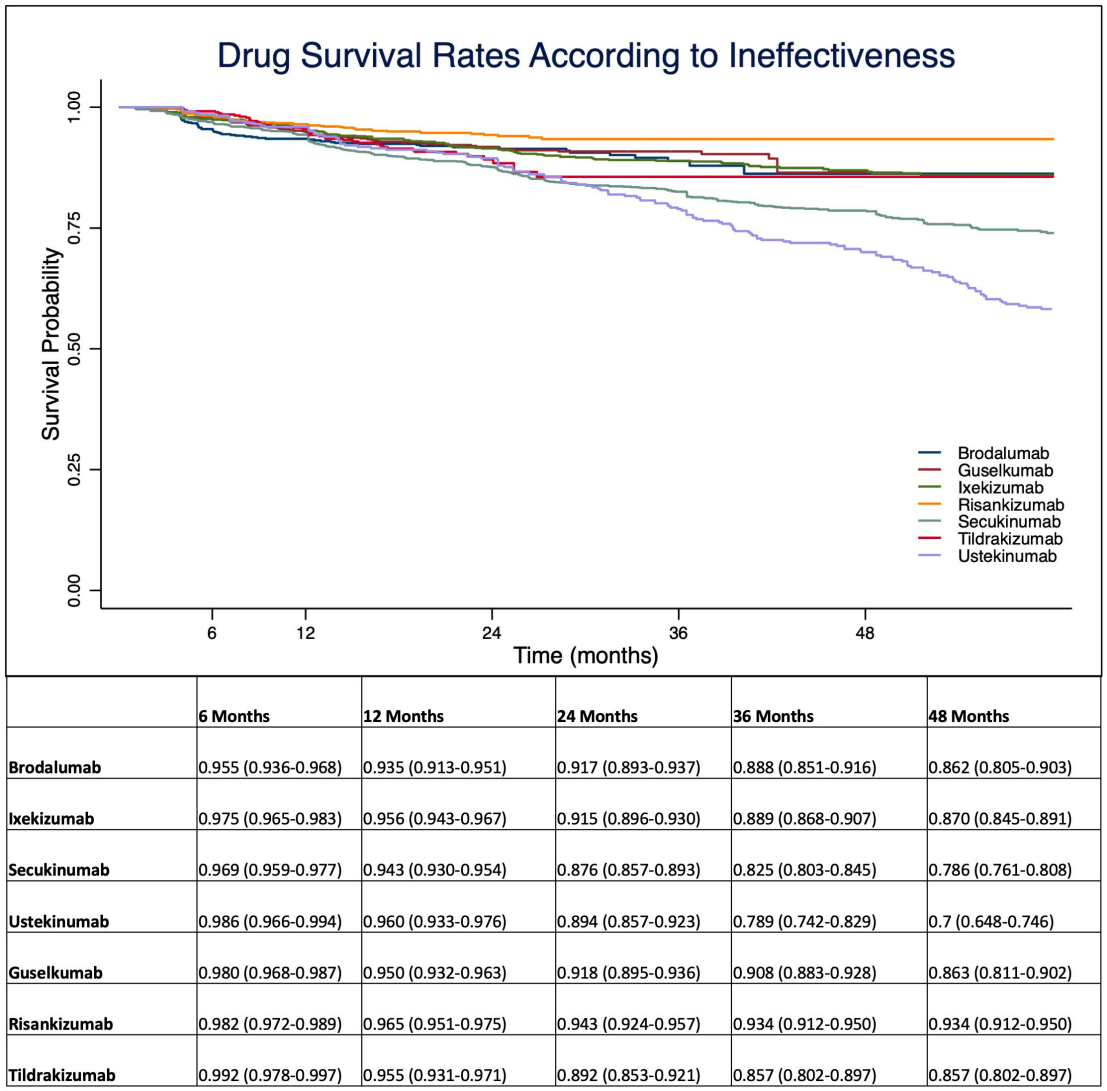
Drug survival rates analyzing only treatment discontinuations due to ineffectiveness. Data are presented as probability with 95% C.I. (Confidence Interval).

At 1-year, anti-IL 12/23 and anti-IL 23 drugs showed a higher drug survival overall, compared with IL-17 inhibitors (**Figure 1**). After four years of follow-up, risankizumab had the highest drug survival overall, as 91.6% of patients were still on treatment with a confidence interval (95% C.I.) of 89.3-93.4. The overall probability of drug survival at four years was comparable for tildrakizumab (83.5%, with a 95% C.I. of 78.1-87.7), ixekizumab (82.6%, 79.9-84.9), guselkumab (82.4%, 76.4-86.5) and brodalumab (81.8%, 75.6-86.6). Secukinumab had a probability of drug survival of 74.7% (72.1-77.0) and ustekinumab of 67.8% (62.6-72.5) (**Figure 1**). We then described the drug survival of all molecules evaluating only the treatment discontinuations due to ineffectiveness, as shown in **Figure 2**. In this analysis, after one year, risankizumab was still the drug with the better performance, as 96.5% of patients (95.1-97.5) were still on treatment after 12 months. It was followed by ustekinumab (96%, 93.3-97.6), ixekizumab (95.6%, 94.3-96.7) and tildrakizumab (95.5%, 93.1-97.1) (**Figure 2**). Regarding this sub-analysis, once again risankizumab emerged as the molecule with the highest drug survival at 4 years (93.4% 91.2-95.0), this time followed by ixekizumab (87%, 84.5-89.1), guselkumab (86.3%, 81.1-90.2), brodalumab (86.2%, 80.5-90.3), tildrakizumab (85.7%, 80.2-89.7), secukinumab (78.6%, 76.1-80.8) and ustekinumab (70%, 64.8-74.6) (**Figure 2**). The log-rank test showed no difference in drug survival in patients with a concomitant PsA (p= 0.41), while bio-naive status was a predictor of better drug survival (p <0.001) compared to patients who had previously failed another biologic.

## 4 Discussion

Given the chronic nature of plaque psoriasis, the treatment persistence of a biological treatment is becoming more and more important in terms of both patients’ quality of life and pharmacoeconomic. The drug survival of treatment represents a marker of both satisfying disease control and good tolerability. As the therapeutic landscape in plaque psoriasis continues to evolve, several drugs have become available to patients. This has allowed for a rapid switch between biologics, making it crucial to understand the treatment persistence of each drug. Our real-world experience, which included one of the largest populations to date, provides more knowledge on the probability of drug survival of different biological treatments for plaque psoriasis, including the most recently approved drugs (tildrakizumab and risankizumab), which were significantly under-represented in previously studies (7–9). In our study, we found higher overall rates of drug survival after four years for IL-23 inhibitors, confirming data from both clinical trials and real-life experiences on the efficacy and safety of this therapeutic class (7–9). In particular, risankizumab was the drug with the highest drug survival overall throughout the study period (**Figure 1**). When evaluating only discontinuation due to ineffectiveness, once again risankizumab emerged as the treatment with the highest drug survival, this time followed by ixekizumab. These findings are consistent with data from recent network meta-analyses that highlighted the high effectiveness profile of ixekizumab and risankizumab (12). In particular, risankizumab has shown high efficacy in clinical trials, with rates of PASI 90 at week 16 of 75.3% and 74.8% in the phase-3 studies UltIMMa 1 and UltIMMa 2, respectively (13). Other clinical trials confirmed the long-term efficacy of risankizumab, as PASI 90 was reached by 86.6% of patients at week 52 in the IMMerge study (14) and by 85.5% of patients in the open-label LIMMitless study after more than 3 years (15). Our findings align with the results of a recent study by Torres et al. (8), which described a higher probability of drug survival for anti-IL-23 treatments, despite a limited follow-up for patients receiving tildrakizumab and risankizumab. In particular, the authors found a cumulative probability of survival at 18 months of 96.4% for risankizumab. The baseline characteristics of our population, also categorized by biological drugs, are comparable with those of other real-world experiences (7–9). As expected in real-world retrospective studies, patients’ populations were not homogeneous among all treatment courses in terms of comorbidities and previous exposure to biological treatments. In our study, consistent with other experiences (7), previous exposure to at least one biological drug had a significant impact on drug discontinuation. On the other hand, the concomitant diagnosis of PsA did not play a significant role in our study.

Nevertheless, our study has a few limitations, which are primarily due to its retrospective nature. Moreover, the different biological treatments were approved in different years, resulting in longer follow-up periods for drugs like ustekinumab and secukinumab and shorter ones for IL-23 inhibitors (in particular, risankizumab and tildrakizumab). In addition, the recent availability of different drugs may have resulted in a higher tendency towards therapeutical switches after a shorter period of treatment in the last couple of years. Being this a retrospective real-world study, different comorbidities were not equally represented among different treatment groups, which could interfere with our results. Also, different ethnicities were not significantly represented in our study, which could limit the generalization of our findings. Further studies should be conducted in the next years to evaluate the treatment persistence of bimekizumab, which was not included in this analysis due to its very recent approval (16).

In conclusion, our experience on more than 5900 treatment courses showed that IL-23 inhibitors, and risankizumab in particular, had a higher probability of drug survival overall during a 4-year follow-up. Risankizumab and ixekizumab were less likely to be discontinued because of ineffectiveness after four years of treatment. Secukinumab and ustekinumab emerged as the drugs with the lowest drug survival overall.

## Data availability statement

The raw data supporting the conclusions of this article will be made available by the authors, without undue reservation.

## Ethics statement

For this retrospective study, institutional review board approval was waived as the study protocol did not deviate from routine clinical practice. In this study, we did not perform any procedure differently from routine clinical practice. All patients included in the study had provided written consent for retrospective analysis of anonymous data collected during routine clinical practice, including demographics and clinical severity scores. This study was conducted in accordance with the Helsinki Declaration of 1964 and its later amendments.

## Author contributions

LG: Conceptualization, Data curation, Formal analysis, Investigation, Visualization, Writing – review & editing. LI: Conceptualization, Data curation, Formal analysis, Investigation, Visualization, Writing – original draft. PM: Conceptualization, Data curation, Methodology, Supervision, Writing – review & editing. AB: Conceptualization, Data curation, Writing – review & editing. FB: Conceptualization, Data curation, Validation, Writing – review & editing. MB: Conceptualization, Data curation, Writing – review & editing. CC: Conceptualization, Data curation, Writing – review & editing. GD: Data curation, Formal analysis, Validation, Writing – review & editing. PD: Conceptualization, Data curation, Writing – review & editing. VD: Data curation, Formal analysis, Writing – review & editing. FG: Data curation, Formal analysis, Writing – review & editing. GG: Conceptualization, Data curation, Methodology, Writing – review & editing. CG: Conceptualization, Data curation, Writing – review & editing. CL: Conceptualization, Data curation, Writing – review & editing. FL: Conceptualization, Data curation, Writing – review & editing. AM: Conceptualization, Data curation, Writing – review & editing. MM: Conceptualization, Data curation, Writing – review & editing. SM: Data curation, Formal analysis, Writing – review & editing. MT: Conceptualization, Data curation, Writing – review & editing. AC: Conceptualization, Data curation, Methodology, Supervision, Validation, Writing – review & editing. AN: Conceptualization, Data curation, Investigation, Methodology, Supervision, Validation, Writing – review & editing.

## References

[1] Griffiths CEM Armstrong AW Gudjonsson JE Barker JNWN. Psoriasis. Lancet (2021) 397(10281):1301–15. doi: 10.1016/S0140-6736(20)32549-6 33812489

[2] Shear NH Betts KA Soliman AM Joshi A Wang Y Zhao J. Comparative safety and benefit-risk profile of biologics and oral treatment for moderate-to-severe plaque psoriasis: A network meta-analysis of clinical trial data. J Am Acad Dermatol (2021) 85(3):572–81. doi: 10.1016/j.jaad.2021.02.057 33631216

[3] Schäkel K Reich K Asadullah K Pinter A Jullien D Weisenseel P. Early disease intervention with guselkumab in psoriasis leads to a higher rate of stable complete skin clearance ('clinical super response'): Week 28 results from the ongoing phase IIIb randomized, double-blind, parallel-group, GUIDE study. J Eur Acad Dermatol Venereol (2023) 37(10):2016–27. doi: 10.1111/jdv.19236 37262309

[4] Gargiulo L Ibba L Malagoli P Amoruso F Argenziano G Balato A. A risankizumab super responder profile identified by long-term real-life observation-IL PSO (ITALIAN LANDSCAPE PSORIASIS). J Eur Acad Dermatol Venereol (2024) 38(1):e113-e116. doi: 10.1111/jdv.19464 37611277

[5] Nast A Smith C Spuls PI Avila Valle G Bata-Csörgö Z Boonen H. EuroGuiDerm Guideline on the systemic treatment of Psoriasis vulgaris - Part 2: specific clinical and comorbid situations. J Eur Acad Dermatol Venereol (2021) 35(2):281–317. doi: 10.1111/jdv.16926 33547728

[6] Gisondi P Fargnoli MC Amerio P Argenziano G Bardazzi F Bianchi L. Italian adaptation of EuroGuiDerm guideline on the systemic treatment of chronic plaque psoriasis. Ital J Dermatol Venerol (2022) 157(Suppl. 1 to No. 1):1–78. doi: 10.23736/S2784-8671.21.07132-2 35262308

[7] Torres T Puig L Vender R Yeung J Carrascosa JM Piaserico S. Drug survival of interleukin (IL)−17 and IL−23 inhibitors for the treatment of psoriasis: A retrospective multi−country, multicentric cohort study. Am J Clin Dermatol (2022) 23(6):891–904. doi: 10.1007/s40257-022-00722-y 35976568

[8] Torres T Puig L Vender R Lynde C Piaserico S Carrascosa JM. Drug survival of IL-12/23, IL-17 and IL-23 inhibitors for psoriasis treatment: A retrospective multi-country, multicentric cohort study. Am J Clin Dermatol (2021) 22(4):567–79. doi: 10.1007/s40257-021-00598-4 33786754

[9] Yiu ZZN Becher G Kirby B Laws P Reynolds NJ Smith CH. Drug Survival Associated With Effectiveness and Safety of Treatment With Guselkumab, Ixekizumab, Secukinumab, Ustekinumab, and Adalimumab in Patients With Psoriasis [published correction appears in JAMA Dermatol. JAMA Dermatol (2022) 158(10):1131–41. doi: 10.1001/jamadermatol.2022.2909 PMC9260644 35791876

[10] Gargiulo L Ibba L Malagoli P Amoruso F Argenziano G Balato A. Brodalumab for the treatment of plaque psoriasis in a real-life setting: a 3 years multicenter retrospective study-IL PSO (Italian landscape psoriasis). Front Med (Lausanne) (2023) 10:1196966. doi: 10.3389/fmed.2023.1196966 37469659 PMC10352451

[11] Taylor W Gladman D Helliwell P Marchesoni A Mease P Mielants H. Classification criteria for psoriatic arthritis: development of new criteria from a large international study. Arthritis Rheumatol (2006) 54(8):2665–73. doi: 10.1002/art.21972 16871531

[12] Sbidian E Chaimani A Guelimi R Garcia-Doval I Hua C Hughes C. Systemic pharmacological treatments for chronic plaque psoriasis: a network meta-analysis. Cochrane Database Syst Rev (2023) 7(7):CD011535. doi: 10.1002/14651858.CD011535.pub 37436070 PMC10337265

[13] Gordon KB Strober B Lebwohl M Augustin M Blauvelt A Poulin Y. Efficacy and safety of risankizumab in moderate-to-severe plaque psoriasis (UltIMMa-1 and UltIMMa-2): results from two double-blind, randomised, placebo-controlled and ustekinumab-controlled phase 3 trials. Lancet (2018) 392(10148):650–61. doi: 10.1016/S0140-6736(18)31713-6 30097359

[14] Warren RB Blauvelt A Poulin Y Beeck S Kelly M Wu T. Efficacy and safety of risankizumab vs. secukinumab in patients with moderate-to-severe plaque psoriasis (IMMerge): results from a phase III, randomized, open-label, efficacy-assessor-blinded clinical trial. Br J Dermatol (2021) 184(1):50–9. doi: 10.1111/bjd.19341 PMC7983954 32594522

[15] Papp KA Lebwohl MG Puig L Ohtsuki M Beissert S Zeng J. Long-term efficacy and safety of risankizumab for the treatment of moderate-to-severe plaque psoriasis: interim analysis of the LIMMitless open-label extension trial beyond 3 years of follow-up. Br J Dermatol (2021) 185(6):1135–45. doi: 10.1111/bjd.20595 PMC9290992 34157132

[16] Gargiulo L Narcisi A Ibba L Balato A Bianchi L Brianti P. Effectiveness and safety of bimekizumab for the treatment of plaque psoriasis: a real-life multicenter study-IL PSO (Italian landscape psoriasis). Front Med (Lausanne) (2023) 10:1243843. doi: 10.3389/fmed.2023.1243843 37614958 PMC10442506

